# Supplementary material for: Cotransporter-mediated water transport underlying cerebrospinal fluid formation
Source: Nat Commun. 2018 Jun 4;9:2167. doi: 10.1038/s41467-018-04677-9 (PMC5986890; doi:10.1038/s41467-018-04677-9)
Supplement: Supplementary file 1 — Supplementary Information [file 41467_2018_4677_MOESM1_ESM.pdf]

Supplementary Information for the manuscript:

Cotransporter-mediated water transport underlying cerebrospinal fluid formation

By Steffensen et al.

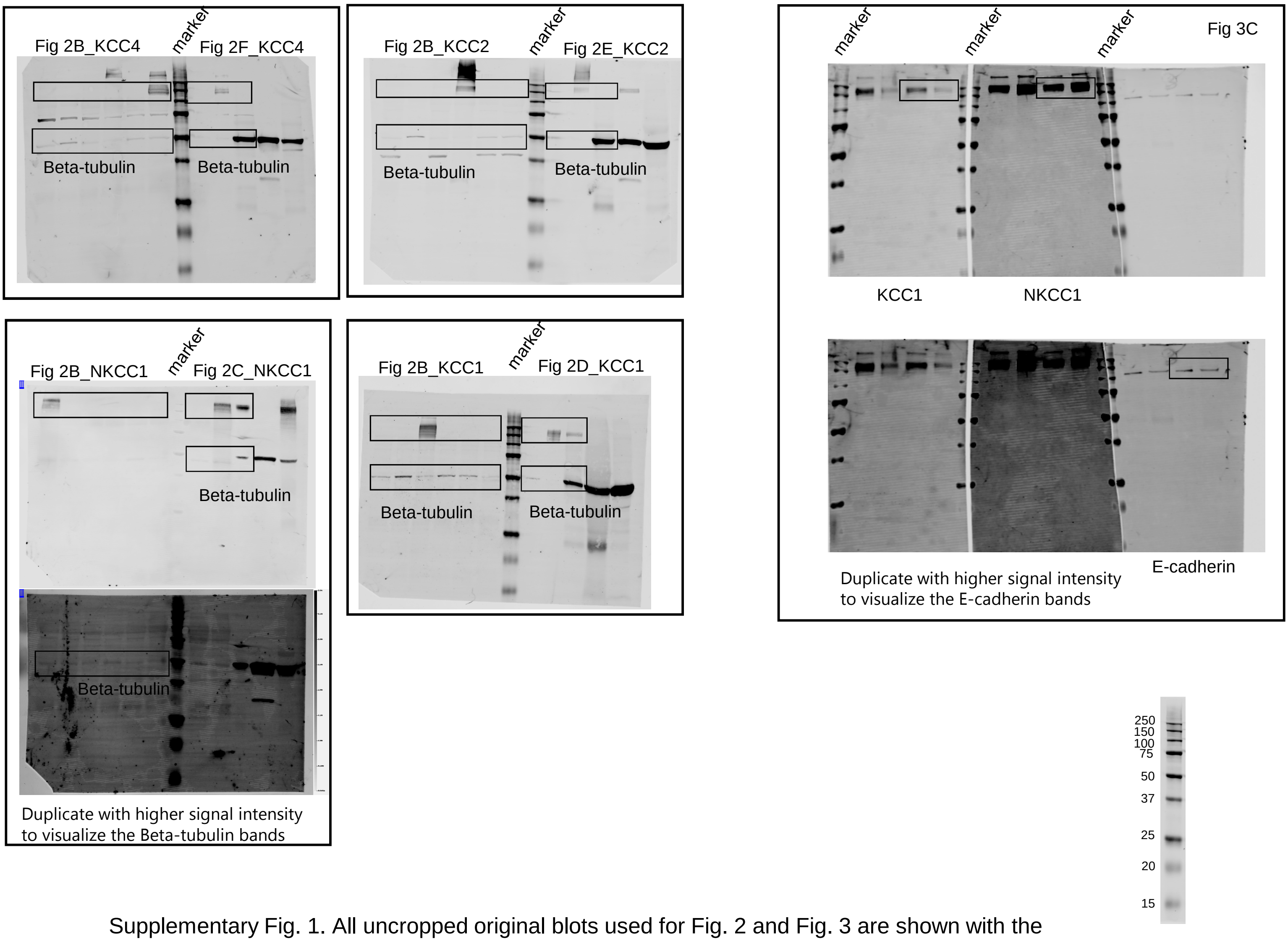

Supplementary Fig. 1. All uncropped original blots used for Fig. 2 and Fig. 3 are shown with the employed parts highlighted with squares. The signals for size markers and protein of interest were acquired in different channels and therefore, when displayed in this manner, the band intensities for the different antibodies can vary. For these cases, we have provided duplicates where one channel is turned up to allow visualization of the respective bands.

The size marker is valid for all western blot images.
